# A heat-shock 20 protein isolated from watermelon (ClHSP22.8) negatively regulates the response of *Arabidopsis* to salt stress via multiple signaling pathways

**DOI:** 10.7717/peerj.10524

**Published:** 2021-03-01

**Authors:** Yanjun He, Yixiu Yao, Lili Li, Yulin Li, Jie Gao, Min Fan

**Affiliations:** 1 Zhejiang Academy of Agricultural Sciences, Institute of Vegetables, Hangzhou, Zhejiang, China; 2 College of Forestry and Horticulture, Xinjiang Agricultural University, Urumqi, Xinjiang, China

**Keywords:** ClHSP22.8, Watermelon (*Citrullus lanatus*), Salt stress, ABA, Arabidopsis

## Abstract

Heat-shock protein 20s (HSP20) were initially shown to play a role during heat shock stress; however, recent data indicated that HSP20 proteins are also involved in abiotic stress in plants. Watermelon is known to be vulnerable to various stressors; however, HSP20 proteins have yet to be investigated and characterized in the watermelon. In a previous study, we identified a negative regulator of salt stress response from watermelon: *ClHSP22.8*, a member of the HSP20 family. Quantitative real-time PCR (qRT-PCR) and promoter::β-glucuronidase (*GUS*) analysis revealed that *ClHSP22.8* was expressed widely in a range of different tissues from the watermelon, but particularly in the roots of 7-day-old seedlings and flowers. Furthermore, qRT-PCR and GUS staining showed that the expression of *ClHSP22.8* was significantly repressed by exogenous abscisic acid (ABA) and salt stress. The over-expression of *ClHSP22.8* in *Arabidopsis* lines resulted in hypersensitivity to ABA and reduced tolerance to salt stress. Furthermore, the expression patterns of key regulators associated with ABA-dependent and independent pathways, and other stress-responsive signaling pathways, were also repressed in transgenic lines that over-expressed *ClHSP22.8*. These results indicated that *ClHSP22.8* is a negative regulator in plant response to salt stress and occurs via ABA-dependent and independent, and other stress-responsive signaling pathways.

## Introduction

Heat-shock proteins (HSPs) act as molecular chaperones and are found in all species of plants. HSPs help to protect their target proteins from denaturation, misfolding, and aggregation, during times of stress (Papsdorf & Richter, 2014; Asea, Kaur & Calderwood, 2016). Previous research has shown that HSPs can be classified into six groups based on molecular weight: HSP100, HSP90, HSP70, HSP60, HSP20 (or small heat-shock protein) and ubiquitin (Asea, Kaur & Calderwood, 2016; Sun & MacRae, 2005). HSP20 is now known to be the largest and best studied family of the HSP families (Basha, O’Neill & Vierling, 2012). Numerous studies have revealed specific roles for plant HSP20 proteins in a range of abiotic stress responses. For example, transgenic plants that over-expressed Heat-shock protein 20s (*HSP20s*) was shown to exhibit enhanced tolerance to heat, including *Arabidopsis*, rice, wheat, maize and Chenopodium (Sun & MacRae, 2005; Sun et al., 2012; Murakami et al., 2004; Charng et al., 2006; Shakeel, Heckathorn & Luthe, 2012; Khurana, Chauhan & Khurana, 2013). Another study showed that the over-expression of *OsHSP20s* in rice led to enhanced tolerance to stress caused by ultraviolet-B radiation, salt, drought, and dehydration (Murakami et al., 2004; Zou et al., 2012; Kaur et al., 2015). Other research studies have shown that the over-expression of *OsHSP17.0* or *OsHSP23.7* led to an improvement in the drought and salt tolerance of rice and that this involved a reduction of membrane damage and increased expression of protective molecules (Zou et al., 2012). Research has shown that *OsHSP18.2* is implicated in seed vigor and longevity and can improve germination and the successful creation of seedlings under abiotic stress (Kaur et al., 2015). Promoter analyses further revealed that the over-expression of *TaHSP26* in wheat could be induced by heat, cold, salt and drought (Chauhan et al., 2012). Furthermore, the over-expression of *TaHSP23.9* in wheat led to an enhancement in the tolerance to heat and salt stresses (Wang et al., 2020). Another study, involving the ectopic expression of *LimHSP16.45* led to an increase in the activities of superoxide dismutase (SOD) and catalase (CAT), thus improving the vigor of seed germination in *Arabidopsis* under salt stress (Mu et al., 2013). The over-expression of *ZmHsp16.9* in tobacco led to an enhancement in the activities of peroxidase (POD), CAT and SOD, and an increase in oxidative stress tolerance (Sun et al., 2012). *HSP20s* have been found to regulate the plant response to salt stress via abscisic acid (ABA) signaling pathways. In *Capsicum annuum*, *CaHsp22.5* was shown to modulate plant ABA signaling and participate in response to salt stress (Li et al., 2018). Other research, carried out in creeping bentgrass, showed that *AsHSP17* and *AsHSP26.8a* mediate ABA-dependent and independent and other stress signaling pathways to negatively regulate plant responses to salt stress (Sun et al., 2016, 2020).

Watermelon (*Citrullus lanatus* L.) is an economically important cucurbit crop that is cultivated across the world. However, it is vulnerable to a variety of adverse environmental conditions (Guo et al., 2013). As one of the most important stressors, salinity stress can lead to serious limitations in the yield and quality of watermelon (Yetr & Uygur, 2009). *HSP20s* are the most abundant HSP sub-type in plants and are known to play important functions in a variety of stress responses (Sun et al., 2012, 2016, 2020; Zou et al., 2012; Wang et al., 2020; Mu et al., 2013; Li et al., 2018). However, we know very little about the specific role of watermelon *HSP20s* with regards to abiotic stress tolerance.

In a previous study, we identified the *HSP20* gene family in watermelon and analyzed their expression patterns in response to different stresses (He et al., 2019). In the present study, we characterized an HSP20 gene (*ClHSP22.8*) from the watermelon. Quantitative real-time PCR (qRT-PCR) and promoter::β-glucuronidase (*GUS*) assays showed that the expression of *ClHSP22.8* was repressed by exogenous ABA and salt treatment. Next, we successfully constructed *Arabidopsis* lines that over-expressed *ClHSP22.8* and used these lines to investigate their sensitivity to ABA and salt tolerance. We analyzed the expression profiles of genes related to ABA and the stress response of plants that over-expressed *ClHSP22.8* under salt treatment. Functional studies of *ClHSP22.8* will not only provide a better understanding of the specific roles of *HSP20s* in the adaption of watermelon to salt stress but may also provide insight into the potential signaling processes in response to stressful conditions.

## Materials and Methods

### Identification of *ClHSP22.8* and bioinformatics analysis

First, we downloaded the amino acid sequence of *ClHSP22.8* from the Cucurbit Genomics Database (accession number: Cla017945). We then used ExPASy (http://web.expasy.org/computepi/) to calculate the molecular weight (MW) and isoelectric point (pI). Conserved domains in the ClHSP22.8 protein were confirmed using the SMART database (http://smart.emblheidelberg.de/). Next, we downloaded the protein sequences of various ClHSP22.8 orthologs in *Arabidopsis*, rice, tomato, soybean, switchgrass, and cucumber, from Phytozome (http://phytozome.jgi.doe.gov/pz/portal.html). Then, we performed phylogenetic analysis, based on the full-length protein sequences, using the MEGA 5.0 program and the neighbor-joining (NJ) method with 1,000 bootstrap replicates (Tamura et al., 2011). Multiple sequence alignment of the predicted peptide sequences of the conserved-crystallin (ACD) domain was then carried out using Clustal X version 1.81 with default parameters (Thompson et al., 1997). The *ClHSP22.8* promoter sequence was also downloaded from the Cucurbit Genomics Database and subsequently analyzed via PlantCARE (http://bioinformatics.psb.ugent.be/webtools/plantcare/html/).

### Plant materials and growth conditions

We used the watermelon advanced inbred line “JJZ-M” for all expression analyses. These plants were grown in a growth chamber in temperature-controlled greenhouses under day/night temperatures of 28/22±1 °C, a light intensity of 200 μmol m^−2^ s^−1^, and a 16-h light/8-h dark photoperiod. Three-week-old watermelon seedlings were used for treatments involving exogenous ABA and salt stress treatments; these treatments involved the seedlings being sprayed with 100 μM of ABA and 200 mM of NaCl, respectively (He et al., 2019). The second true leaf on each plant was sampled at time 0 (control) and then again at 1, 4 and 12 h after treatment. *Arabidopsis thaliana* ecotype “Columbia” (wild type, WT) plants were used for the construction of transgenic plants. These plants were kept at 24/22 °C (16 h-day/8 h-night) with 65% relative humidity to yield transgenic lines. Both transgenic and WT plants were cultivated under the same growth conditions.

### Quantitative real-time PCR

Total RNA was isolated from samples of both watermelon and *Arabidopsis*. Reverse transcription was then performed using the PrimeScript RT reagent kit (Takara, China) in accordance with the manufacturer’s instructions; for each sample, approximately 1 μg of total RNA was reverse transcribed into cDNA. qRT-PCR reactions were performed on a CFX96 Real Time PCR System (Bio-Rad, USA) using the following cycle conditions: 30 s at 95 °C; followed by 40 cycles of 5 s at 95 °C, and 45 s at 55 °C; this was followed by 1 cycle of 1 min at 95 °C, 30 s at 50 °C and 30 s at 95 °C. Two biological and three technical replicates were carried out for each sample; these reactions involved a reaction volume of 15 μL and the SYBR Premix Ex Taq kit (Toyobo, Japan). We used the watermelon β-*actin* gene and the *Arabidopsis ACTIN2* gene as reference sequences for primer design (Table S1) and relative gene expression was calculated using the 2^−ΔΔCt^ method.

### Promoter assay by GUS histochemical staining

In order to investigate the tissue-specific expression of *ClHSP22.8*, we amplified a 1601 bp upstream promoter sequence using specific primer pairs (Table S1). We then ligated this fragment with the pBI101 plasmid vector that could be subsequently transformed into *Arabidopsis*. The reporter construct containing the GUS reporter gene driven by the *ClHSP22.8* promoter region was named ProClHSP22.8::*GUS* (Fig. S1). Transgenic *Arabidopsis* seedlings were obtained using the floral dip method (Xiuren et al., 2006). Transgenic *Arabidopsis* seeds were screened using 100 mg L^−1^ kanamycin (KanR). Positive transgenic plants were identified by GUS histochemical staining. T3 transgenic lines were also screened and harvested for further phenotypic observation.

The 7-day-old *Arabidopsis* transgenic seedlings created from the T3-generation grown on 1/2 MS medium were transferred to 1/2 MS medium with and without 100 μM of ABA and 200 mM of NaCl. After 24 h, the transgenic seedlings were GUS stained using a GUS Histochemical Staining Kit (O’BioLab, Beijing, China) in accordance with the manufacturer’s guidelines. After removing chlorophyll with 70% ethanol, we analyzed the seedlings and acquired typical digital images using a stereomicroscope (STEMI SV11, Zeiss, Jena, Germany).

### Plasmid construction and generation of transgenic plants

The coding sequence (CDS) of *ClHSP22.8* was amplified using a pair of specific primers: *ClHSP22.8*-F and *ClHSP22.8*-R (Table S1). The amplicons produced by PCR were subsequently digested and ligated into the pBI121 vector (Fig. S1). Subsequently, the pBI121-p35S::*ClHSP22.8* vector was transformed into *Arabidopsis* using the floral dip method. Transgenic *Arabidopsis* lines were confirmed by PCR using two specific primers: HSP22.8-S and GUS-A (Fig. S2). The progenies of these plants were screened using 100 mg L^−1^ KanR, as described earlier. As a result, four independent homozygous transgenic lines were created; we named these OE22.8-1, OE22.8-2, OE22.8-3 and OE22.8-4. T3 transgenic lines were screened and harvested for further phenotypic observation.

### ABA and salt tolerance in transgenic *Arabidopsis* lines

The T3-generation transgenic lines (OE22.8-1–OE22.8-4) were grown with WT seedlings on 1/2 MS medium with or without 100 μM of ABA and 200 mM of NaCl, respectively. After 7 days, we photographed these plants so that we had a record of their relative phenotypes. The growth status and root length of two-week-old plants were measured, and samples were taken for qRT-PCR. Three independent biological replicates were analyzed; each replicate involved over 30 seedlings.

### Statistical analysis

Data were analyzed by a two-tailed Student’s *t*-test or by one-way analysis of variance (ANOVA) using SPSS version 18.0 (IBM, Chicago, IL, USA). *P < 0.05 and **P < 0.01 were considered to be significant and highly significant, respectively.

## Results

### Isolation and bioinformatics analysis of *ClHSP22.8*

**Figure 1:**
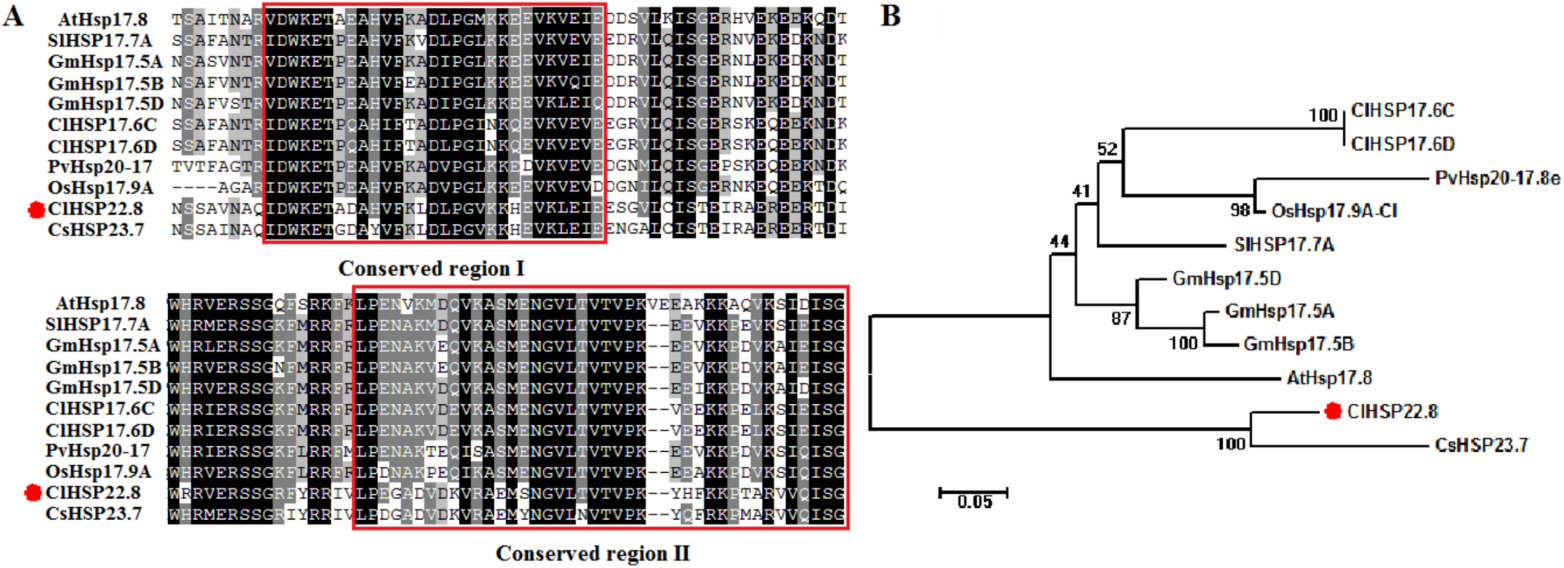
Phylogenetic and amino acid sequence analysis of ClHSP22.8 orthologs in various species. (A) Amino acid sequence alignment of α-crystallin ACD/HSP20 domain from *Arabidopsis* (At), rice (Os), tomato (Sl), soybean (Gm), switchgrass (Pv), cucumber (Cs) and watermelon (Cl). Conserved region I and II were indicated by red boxes. (B) Phylogenetic analysis of ClHSP22.8 protein orthologs from these orthologs. Phylogenetic analysis based on full-length protein sequences was performed using the MEGA 5.0 program by the neighbor-joining (NJ) method with 1,000 bootstrap replicates.

The length of the full-length coding sequence (CDS) for the *ClHSP22.8* gene was 582 bp and coded for a protein containing 193 amino acids. The isoelectric point (pI) of the protein was 7.76, and the molecular weight (MW) was 22.85 kDa (Table S1). *ClHSP22.8* on Chr 10 was mapped to a segmentally duplicated region that was shared with *ClHSP16*, *ClHSP17.6C* and *ClHSP17.6D*, respectively (Table S1). The ClHSP22.8 protein shared a conserved α-crystallin ACD/HSP20 domain (thus showing conservation of region I and II) with its orthologs (Fig. 1A). Phylogenetic analysis further showed that ClHSP22.8 and CsHSP23.7 in the cucumber formed a separate branch that was distant from a range of other orthologs from *Arabidopsis*, rice, tomato, soybean and switchgrass (Fig. 1B).

### Spatial and temporal expression of *ClHSP22.8* in watermelon

**Figure 2:**
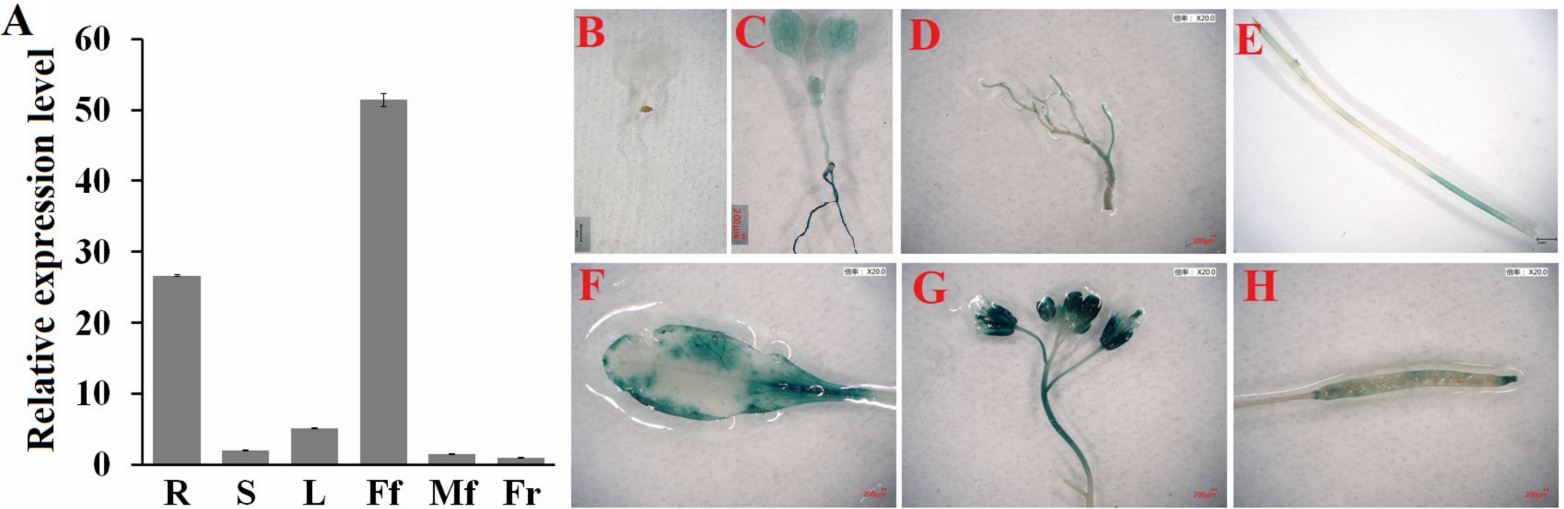
Spatial and temporal expression patterns of *ClHSP22.8*. (A) *ClHSP22.8* expression levels in root (R), stem (S), leaf (L), female flower (Ff), male flower (Mf) and fruit (Fr) of watermelon via quantitative real-time PCR analysis (qRT-PCR). Histochemical GUS assays of ProClHSP22.8::*GUS* in *Arabidopsis*. GUS protein was expressed in 7-day-old seedlings with *GUS*-PBI101 (mock) (B) and ProClHSP22.8:: *GUS* (C). (D–H) represents the GUS signal was expressed in root (D), stem (E), leaf (F), flower (G) and silique (H) of adult *Arabidopsis* plants.

Next, we determined the spatial and temporal expression profiles of *ClHSP22.8* via qRT-PCR. Data indicated that *ClHSP22.8* was widely expressed across a range of different tissues in the watermelon and showed the lowest level in the fruit; higher levels of expression were observed in female flowers and roots (51.37- and 26.63-fold compared to that in the fruit) (Fig. 2A). In order to further characterize the tissue-specific expression of the *ClHSP22.8* gene, we amplified a 1,601 bp fragment from a region that was upstream of the ATG start codon in the *ClHSP22.8* gene. We then transfected this fragment into *Arabidopsis* in order to drive β-glucuronidase (*GUS*) gene expression (Fig. S1; Figs. 2B–2G). Results from GUS histochemical staining indicated that the GUS protein was expressed at the highest levels in roots; the next highest level of expression was seen in the cotyledons, especially in the leaf vein. Signals were also detected in the vascular tissue of hypocotyls in 7-day-old *Arabidopsis* seedlings (Fig. 2B). In adult *Arabidopsis* plants, the GUS signal was expressed at the highest levels in flowers, followed by leaves. Only weak signals were detected in the stem, silique and roots (Figs. 2C–2G).

### Response patterns of watermelon *ClHSP22.8* to ABA and salt stress

**Figure 3:**
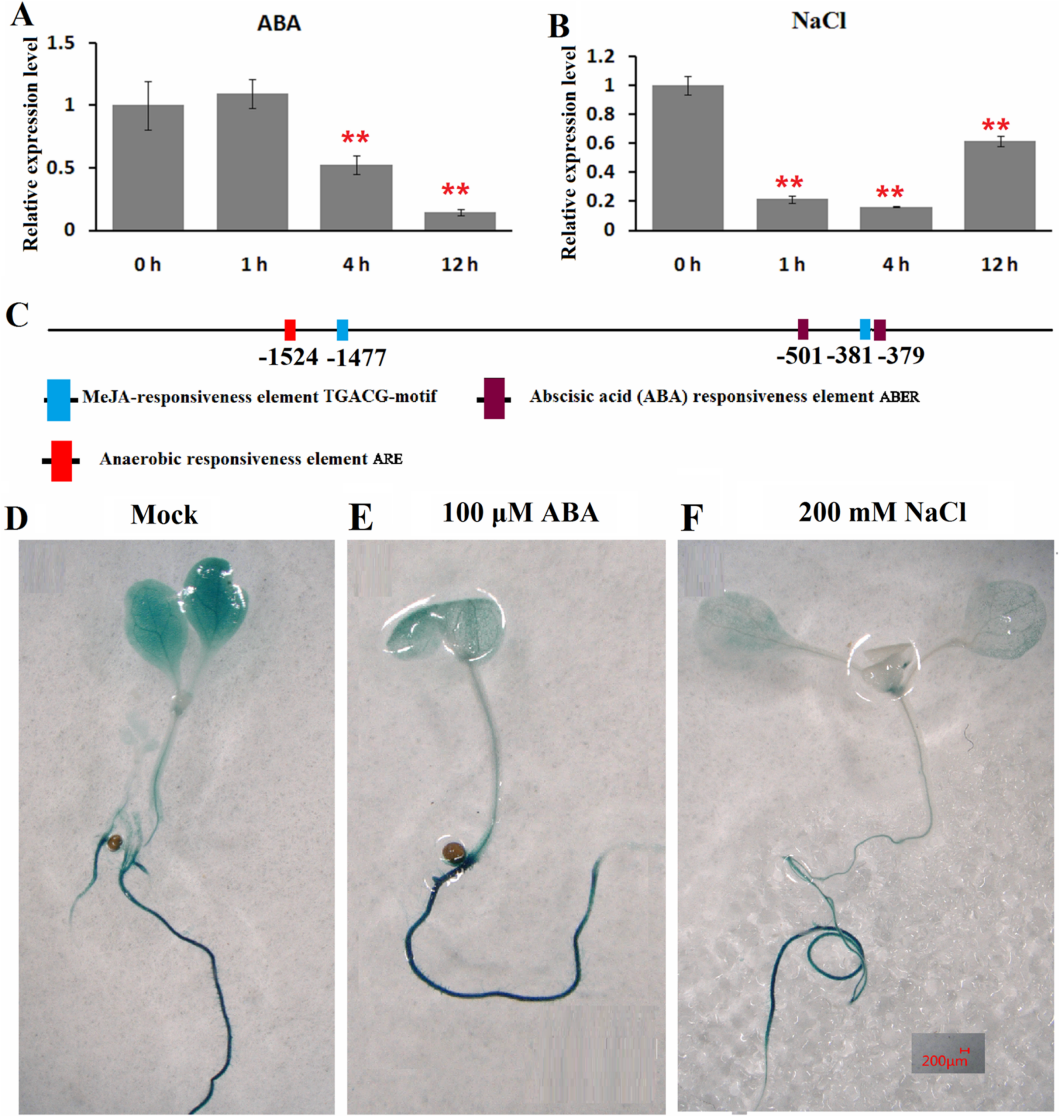
Expression profiles of *ClHSP22.8* in response to abscisic acid (ABA) and salt stress. *ClHSP22.8* expression levels in watermelon leaves exposed to 100 μM ABA (A) and 200 mM NaCl (B) at 0, 1, 4 and 12 h via qRT-PCR. The asterisks on the top of the columns indicate significant differences from the value at 0 h. **P < 0.01 (C) *Cis*-elements analysis of *ClHSP22.8* promoter sequence. (D) Histochemical GUS assays of 7-day-old transgenic seedlings with empty vector. (E and F) Histochemical GUS assays of 7-day-old transgenic seedlings with ProClHSP22.8::*GUS* exposed to ABA and NaCl for 24 h.

The expression patterns of *ClHSP22.8* in response to ABA and salt stress were detected by qRT-PCR in watermelon leaves at four different timepoints (0, 1, 4 and 12 h). Results indicated that *ClHSP22.8* expression was obviously downregulated by 0.52-and 0.14-fold at 4 and 12 h after ABA treatment, respectively (Fig. 3A). Similarly, the expression of *ClHSP22.8* was significantly reduced by salt stress and reached a minimum at 4 h (0.16-fold), although the level of reduction appears to weaken at 12 h (0.61-fold) (Fig. 3B).

*Cis*-acting elements of the *ClHSP22.8* promoter sequence were subsequently analyzed via PlantCARE website. Two ABA responsiveness elements (ABREs), one anaerobic responsiveness element (ARE) and two MeJA-responsiveness elements (TGACG-motifs) were identified (Fig. 3C); Two ABRE elements were located at −501 and −379 bp; the ARE element was located at −1,524 bp; and two MeJA-responsiveness elements were located at −1,477 and −381 bp. We were not able to detect an HSE motif in the promoter.

ProClHSP22.8::*GUS* analysis showed that there were no significant differences in terms of GUS staining in roots of 7-day-old seedlings in response to ABA and salt treatment at 24 h when compared with untreated seedlings, although there was a significant reduction in the leaves (Figs. 3D–3F).

### Overexpression of *ClHSP22.8* conferred ABA sensitivity to *Arabidopsis*

**Figure 4:**
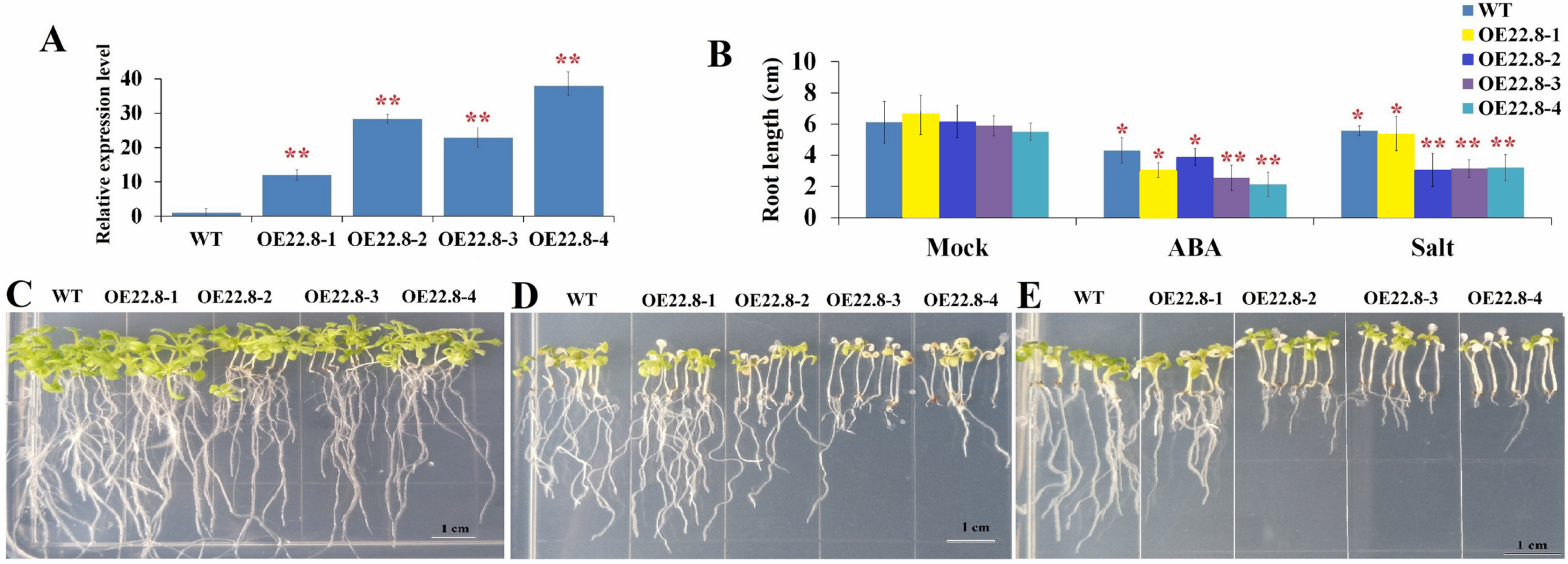
Seedling assay of *ClHSP22.8*-overexpressing lines and wild type (WT) in response to ABA and NaCl treatment. (A) Relative gene expression of *ClHSP22.8* in different overexpression *Arabidopsis* transgenic lines OE22.8-1 to OE22.8-4. (B) The root length of the OE22.8 transgenic and WT plants in the presence of 100 mM ABA and 200 mM NaCl for 7 days after germination, respectively. Growth of the transgenic and WT plants under normal condition (C) and in the presence of 100 mM ABA (D) and 200 mM NaCl (E) for 7 days after germination. * and ** represent significant differences between WT and OE22.8 lines at values of *P* < 0.05 and *P* < 0.01, respectively, as determined by Student’s *t*-test.

We constructed *Arabidopsis* lines that over-expressed *ClHSP22.8* and screened these lines by both PCR and qRT-PCR (Fig. S2; Fig. 4). Compared to the WT as a control, we found that the *ClHSP22.8* gene was significantly over-expressed by 8.60-, 20.32-, 16.38- and 27.20-fold in the OE22.8-1, OE22.8-2, OE22.8-3 and OE22.8-4 lines, respectively (Fig. 4A). To determine the ABA sensitivity of the lines that over-expressed *ClHSP22.8*, we grew the OE22.8 lines, along with the WT plants, on 1/2 MS medium with 100 μM of ABA. After 7 days, we found that the root growth of the WT plants had decreased by 29.61% in response to exogenous ABA treatment when compared with untreated WT seedlings (defined as mock). However, we found that root growth in the OE22.8-1, OE22.8-2, OE22.8-3 and OE22.8-4 seedlings was seriously repressed, by 36.88–61.08%, when compared to the WT plants (Figs. 4B–4E). This data indicated that plants that over-expressed *ClHSP22.8* were more sensitive to ABA treatment.

### Overexpression of *ClHSP22.8* reduced tolerance to salt in transgenic *Arabidopsis*

In order to further investigate the specific roles of *ClHSP22.8* in response to salt stress, we subjected 7-day-old seedlings that over-expressed *ClHSP22.8* (OE22.8-1, OE22.8-2 and OE22.8-4 lines) and WT plants to 200 mM NaCl. After 7 days, the seedlings that over-expressed *ClHSP22.8* showed more chlorosis and stunted phenotypes; their primary root lengths were also more significantly reduced than the WT plants (Fig. 4). Compared with mock seedlings, we found that the root length in WT plants after salt treatment was reduced by 8.87% in response to salt treatment. In contrast, the OE22.8 lines showed a more serious reduction in root length (19.35–50.43%). This data indicated that *ClHSP22.8* negatively regulates plant salinity stress response.

### Some ABA- and stress-related genes were repressed by over-expression of *ClHSP22.8* under salt stress

**Figure 5:**
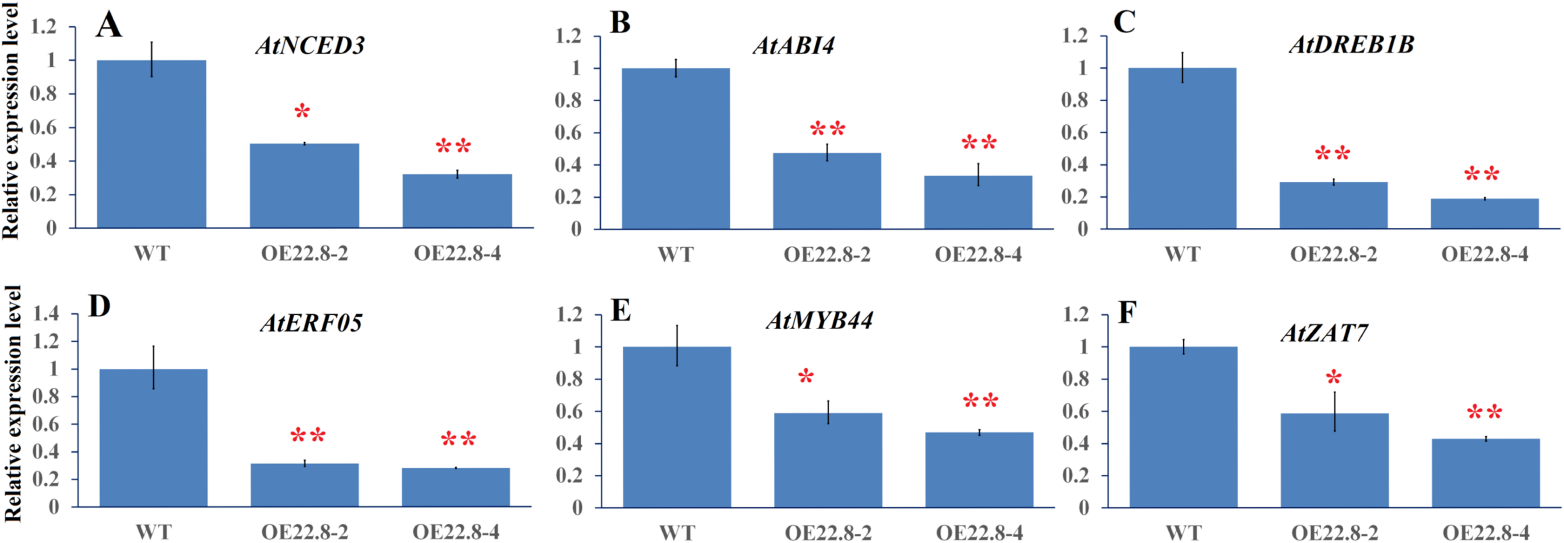
Expression profiles of ABA- and stress-related genes in WT and ClHSP22.8 overexpressing *Arabidopsis* plants. (A–F) Represent the expression profiles of *Arabidopsis* 9-cis epoxycarotenoid dioxygenase 3 (AtNCED3), ABA insensitive 4 (AtABI4), *Arabidopsis* dehydration-responsive element-binding protein 1B (AtDREB1B), ethylene response factor 05 (AtERF05), myb domain protein 44 (AtMYB44), and zinc finger protein (AtZAT7) respectively. * and ** represent significant differences from the control at values of *P* < 0.05 and *P* < 0.01, respectively, as determined by Student’s *t*-test.

Finally, we used qRT-PCR to investigate the expression profiles of several representative genes that are involved in ABA biosynthesis and signaling and stress-responsive transcription factors (TFs), including *Arabidopsis* 9-cis epoxycarotenoid dioxygenase 3 (*AtNCED3*), ABA insensitive 4 (*AtABI4*), ethylene response factor 05 (*AtERF05*), *Arabidopsis* dehydration-responsive element-binding protein 1B (*AtDREB1B*), zinc finger protein (*AtZAT7*) and myb domain protein 44 (*AtMYB44*). In the WT plants, we found that *AtNCED3*, *AtABI4*, and *AtERF05* were repressed by 0.28–0.57-fold, while *AtDREB1B*, *AtMYB44* and *AtZAT7*, were induced by 2.22–4.35-fold after salt treatment (Fig. S3). These results indicate that these six genes are salt-responsive genes. Furthermore, the expressions of these six genes were significantly repressed in the OE22.8-2 (from 0.47- to 0.58-fold) and OE22.8-4 (from 0.32- to 0.46-fold) lines compared to that in the WT plants (Fig. 5). This data indicated that the overexpression of *ClHSP22.8* could repress the expression of these stress-responsive genes in *Arabidopsis*.

**Figure 6:**
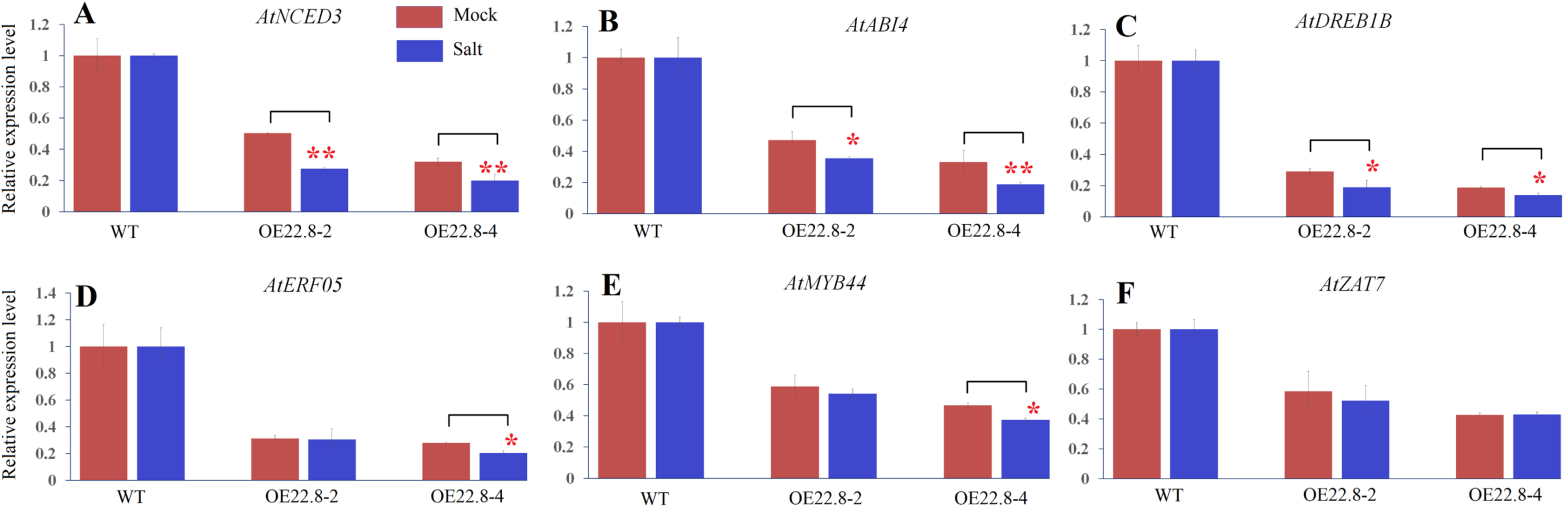
Expression profiles of ABA- and stress-related genes in three-week-old WT and ClHSP22.8 overexpressing Arabidopsis plants 24 h after salt treatment. (A–F) Represent the expression profiles of AtNCED3, AtABI4, AtDREB1B, AtERF05, AtMYB44, and AtZAT7 respectively. The changes observed in WT were normalized. * and ** represent significant differences between the mock- and salt-treatment OE22.8 plants at values of *P* < 0.05 and *P* < 0.01, respectively, as determined by Student’s *t*-test.

In order to confirm whether the reduction/elevation in expression was more pronounced in the OE22.8 lines when treated with 200 mM NaCl, we normalized the changes observed in WT plants and then checked if the OE22.8 lines showed statistically significant differences in terms of gene expression (Fig. 6). We observed a significant reduction in the expression of *AtNCED3*, *AtABI4* and *AtDREB1B* in the OE22.8-2 and OE22.8-4 lines after salt treatment. The expression levels of *AtERF05* and *AtMYB44* were obviously repressed, but only in the OE22.8-4 line. There were no significant differences in the expression levels of *AtZAT7* in the OE22.8 lines after salt treatment. These results indicate that all of the detected genes, except *AtZAT7*, might represent target genes for *ClHSP22.8*-mediated repression in response to salt stress.

## Discussion

Salinity is an important environmental stress factor and can have a severe effect on the growth and development of plants. Consequently, salt stress is a growing problem for global agricultural production (Abbasi et al., 2016). Watermelon (*Citrullus lanatus*) is a salt-sensitive crop and may help us to engineer more salt-tolerant varieties so that we can investigate the core salt-tolerance mechanisms in this fruit (Yetr & Uygur, 2009). As the largest family, and most well studied HSP, the HSP20 family of proteins is ATP-independent and generally assemble into large oligomers that can protect other proteins from denaturation and aggregation (Papsdorf & Richter, 2014; Asea, Kaur & Calderwood, 2016). An increasing body of evidence has shown that *HSP20s* regulate the responses of plants to environmental changes and thus allow plants to survive adverse conditions (Sun et al., 2012, 2016, 2020; Murakami et al., 2004; Charng et al., 2006; Shakeel, Heckathorn & Luthe, 2012; Khurana, Chauhan & Khurana, 2013; Zou et al., 2012; Kaur et al., 2015; Chauhan et al., 2012; Wang et al., 2020; Mu et al., 2013; Li et al., 2018). In a previous study, we identified *HSP20* genes within the genome of watermelon and found that *HSP20s* in plants can be divided into 18 subfamilies (He et al., 2019). The largest group is the nucleocytoplasmic (C)-located HSP20s; this group features 13 subfamilies that exhibit functional redundancy and divergence (He et al., 2019; Guo et al., 2020; Kim et al., 2011). As a member of the CIX subfamily, ClHSP22.8 has a ACD domain, agglomerates into granules in the cytoplasm, and existes as larger oligomers in vivo as expected (He et al., 2019). Gene duplication events are major sources of new gene functions (Francino, 2005). *ClHSP22.8* was duplicated with *ClHSP17.6C*, *ClHSP17.6D*, and *ClHSP16*, from the CI subfamily, but it does not have a close relationship with the duplicates, or homologs in other species, except for CsHSP23.7 in cucumber (Fig. 1B; Table S1) (He et al., 2019). Notably, we found *ClHSP22.8* could not be induced by heat but was significantly repressed by salt stress (Fig. 3B), which was different from a typical HSP20 that exhibited rapid and significant upregulation under heat stress (Papsdorf & Richter, 2014; Asea, Kaur & Calderwood, 2016; He et al., 2019). Therefore, we consider that *ClHSP22.8* probably evolved new functionality in stress responses following the gene duplication event (Francino, 2005).

In order to verify the precise functional role of *ClHSP22.8* in salt stress response, we created lines of *Arabidopsis* that over-expressed *ClHSP22.8* and found that these overexpression lines exhibited shorter roots and more yellow leaves under salt stress. Thus, these results indicate that *ClHSP22.8* negatively regulates the salinity tolerance of *Arabidopsis* (Fig. 4). In this study, we used qRT-PCR and promoter::*GUS* analysis analysis and found that *ClHSP22.8* was clearly repressed by salt treatment (Fig. 3B). The transcript abundance of *ClHSP22.8* in response to salt stress reached the lowest level in the first 4 h. The response pattern of *ClHSP22.8* that rapidly and sharply responded to salt stress in a short time and then had slight variations, was similar to quite a few *HSP20s* such as *TaHSP23.9* (Wang et al., 2020), *OsHSP20* (Guo et al., 2020), *AsHSP17* (Sun et al., 2016) and so on. The response pattern of *HSP20s* probably is a kind of mechanism for plants rapidly adapt to salt stress.

*HSP20s* are known to modulate the multiple signaling pathway so as to regulate the plant’s response to salt stress. Most *HSP20s* have been reported to play positive roles in regulating plant tolerance to salt, including maize *ZmHsp16.9* (Sun et al., 2012), rice *OsHSP17.0* and *OsHSP23.7* (Zou et al., 2012), wheat *TaHSP23.9* (Wang et al., 2020), David Lily *LimHSP16.45* (Mu et al., 2013) and sweet pepper *CaHsp22.5* (Li et al., 2018). However, recent work, a few studies about the negative effect of *HSP20s* on plant response to salt stress have been reported. The over-expression of *AsHSP17* or *AsHSP26.8* in plants led to the direct repression of the vast majority of stress-responsive genes involved in plant photosynthesis, ABA-dependent and ABA-independent pathways, and some other stress response pathways, thus led to reduced levels of salt tolerance (Sun et al., 2016, 2020), but the negative regulation mechanisms of *HSP20s* in salt stress responses remain to be fully unraveled. Similar to *AsHSP17* and *AsHSP26.8* (Sun et al., 2016, 2020), *ClHSP22.8* also plays negative roles in terms of salt response involved in ABA signaling pathway. ABA responsiveness element (ABRE) and anaerobic response element (ARE) *cis*-elements can be recognized by AREB/ABF and MYB transcription factors, respectively (Banerjee & Roychoudhury, 2017; Fujita et al., 2011). The two *cis*-elements are necessary for ABA- and anaerobic-responsive gene expression (Banerjee & Roychoudhury, 2017; Fujita et al., 2011). In the present study, two ABRE and one ARE elements were identified in the *ClHSP22.8* promoter (Fig. 3C). And *ClHSP22.8* was significantly repressed by the exogenous ABA via qRT-PCR and promoter::*GUS* analysis (Figs. 3A, 3D and 3E). Above results indicate that *ClHSP22.8* respond to salinity stress in a negative manner and that this response involved the ABA mediated signaling pathway.

To further illustrate the regulatory mechanism of *ClHSP22.8* under salt stress, we used qRT-PCR to determine the expression patterns of six ABA- and stress-related genes in response to salt stress; results demonstrated that all of these detected genes could obviously respond to salt stress which *AtNCED3*, *AtABI4*, and *AtERF05* were obviously repressed while *AtDREB1B*, *AtMYB44*, and *AtZAT7* were induced by salt stress (Fig. S3). Meanwhile, studies found that over 90% differentially expressed genes (DEGs) in *AsHSP17* and *AsHSP26.8* overexpressed lines were down-regulated (Sun et al., 2016, 2020). Similarly, all of the detected genes in this study were significantly repressed by overexpression of *ClHSP22.8*. These results indicate all of the six detected genes are salt-responsive genes and repressed by *ClHSP22.8*.

ABA-dependent pathways is an important mechanism in adaptation to salt stress and affacts plant salt stress response and tolerance (Roychoudhury, Paul & Basu, 2013; Yoshida, Mogami & Yamaguchi-Shinozaki, 2014). ABA biosynthesis and signalling and some stress-responsive transcription factors involved in ABA signalling were key regulators in ABA-dependent pathway. Among the detected genes in this study, *AtNCED3* encodes a rate-limiting enzyme that plays a role in ABA biosynthesis and *ABI4* is a key regulator of the ABA-dependent pathway (Roychoudhury, Paul & Basu, 2013; Yoshida, Mogami & Yamaguchi-Shinozaki, 2014). Their expression were significantly repressed in OE22.8 lines after normalized the changes observed in WT plants (Fig. 6). So the over-expression of *ClHSP22.8* probably resulted in lower levels of ABA and enhanced ABA sensitivity by repressing the expression of *AtNCED3* and *ABI4* transcripts in response to salt stress. Besides, some *MYB* and *ZAT* transcription factors have been implicated in the plant response to abiotic stress and ABA sensitivity (Ciftci-Yilmaz et al., 2007; Nguyen & Cheong, 2018; Yu et al., 2017; Wei et al., 2017; Seo et al., 2012; Persak & Pitzschke, 2014). *AtMYB44* was involved in ABA-dependent signaling pathways that regulate stress adaption and confer plant tolerance to salt stress (Nguyen & Cheong, 2018; Seo et al., 2012; Persak & Pitzschke, 2014). And the constitutive expression of *AtZAT7* suppressed growth and enhanced salt tolerance in transgenic *Arabidopsis* plants (Ciftci-Yilmaz et al., 2007). *AtMYB44* and *AtZAT7* have both been shown to be regulated by *HSP20s* in response to salt stress (Sun et al., 2016, 2020). In this study, we found that the expression of *AtMYB44* and *AtZAT7* can be obviously induced by salt in WT plants but the reduction of *AtMYB44* is more pronounced in the OE22.8-4 plants (Fig. S3; Fig. 6). These results indicated that *ClHSP22.8* participates in the response to salt stress via an ABA-dependent pathway.

Our data suggested *ClHSP22.8* also modulated the response to salt stress via an ABA-independent signaling pathway. Previous work has shown that *AtDERB1B* and *AtERF05* participate in ABA-independent pathway (Sun et al., 2020; Yoshida, Mogami & Yamaguchi-Shinozaki, 2014); and we found *AtDERB1B* and *AtERF05* were both *ClHSP22.8*-regulated salt responsive genes. The expression of *AtDERB1B* were obviously induced while *AtERF05* were repressed by salt stress in WT plants (Fig. S3). And they were both significantly repressed in OE22.8 lines (Fig. 5). In further, the two genes were significantly repressed by salt stress in OE22.8 lines after normalizing the effects shown in WT plants (Fig. 6).

**Figure 7:**
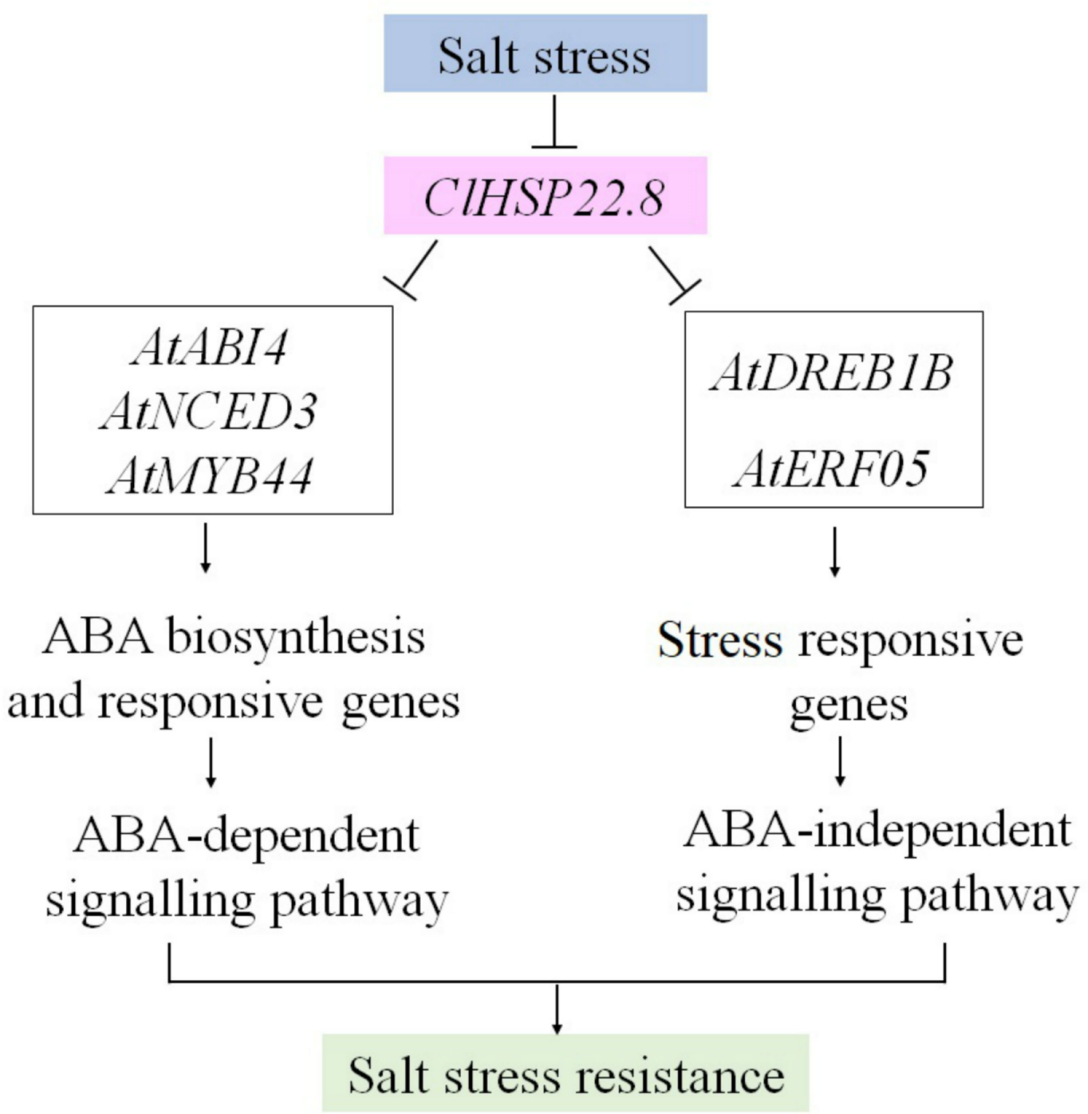
A proposed model for the roles of *ClHSP22.8* in salt stress resistance in *Arabidopsis*. *ClHSP22.8* is involved in ABA biosynthesis, ABA-dependent and independent, and other stress responsive signaling pathways to modulate plant response to salt stress. Abbreviations: *AtNCED3*, *Arabidopsis* 9-cis epoxycarotenoid dioxygenase 3; *AtABI4*, *Arabidopsis* ABA insensitive 4; *AtERF05*, ethylene response factor 05; *AtDREB1B*, *Arabidopsis* dehydration-responsive element-binding protein 1B; *AtMYB44*, *Arabidopsis* myb domain protein 44. Arrows indicate positive regulation, whereas lines ending with a bar indicate negative regulation.

Collectively, these results imply that, as a negative regulator of salt stress, *ClHSP22.8* may be repressed to an appropriate level in protecting plants from salt stress. However, when *ClHSP22.8* was overexpressed in *Arabidopsis*, some genes involved in ABA-dependent (*AtNCED3* and *ABI4*) and ABA-independent (*AtDERB1B* and *AtERF05*) signaling pathways, and stress-responsive TF (*AtMYB44*) in ABA signalling were repressed, and then the salt stress response regulatory network was negatively impacted as outlined in Fig. 7. Our data provides further understanding of the specific roles of *HSP20s* in the watermelon in terms of the response to abiotic stress.

## Conclusions

In summary, we identified an *HSP20* gene, *ClHSP22.8*, and demonstrated that this gene plays important roles in the salt response. Analyses involving both qRT-PCR and promoter::*GUS* analysis indicated that the expression of *ClHSP22.8* in *Arabidopsis* could be repressed by exogenous ABA and salt stress. Furthermore, the over-expression of *ClHSP22.8* repressed some genes that are known to be involved in ABA-dependent and independent signaling pathways, and other stress-responsive pathways, thus leading to an enhanced level of plant sensitivity to ABA and a reduced tolerance to salt stress. Our study provides a better understanding of the specific roles of *HSP20s* in watermelon with regards to the abiotic stress response and suggests that *ClHSP22.8* may be a valuable gene to consider when cultivating watermelons.

## Supplemental Information

10.7717/peerj.10524/supp-1 Supplemental Information 1 Schematic diagram of the *ClHSP22.8* gene expression construct. (A) p35S:: *ClHSP22.8*/p35S-KanR. The *ClHSP22.8* gene (coding sequence only), and a resistance gene, kanamycin (KanR), were both under the control of the CaMV35S promoter. RB, right border; LB, left border (B) Schematic diagram of the ProClHSP22.8::*GUS*-PBI101 vector.

10.7717/peerj.10524/supp-2 Supplemental Information 2 PCR analysis of *ClHSP22.8* in *Arabidopsis* wild type (WT) and OE22.8 transgenic lines. Lanes 1 to 7 indicate the DNA marker, WT, p35S::*ClHSP22.8* vector, and OE22.8-1 to OE22.8-4, respectively.

10.7717/peerj.10524/supp-3 Supplemental Information 3 Expression patterns of ABA- and stress-related genes in response salt stress via quantitative real-time PCR (qRT-PCR). The expression patterns of *Arabidopsis* 9-cis epoxycarotenoid dioxygenase 3 (*AtNCED3*), ABA insensitive 4 (*ABI4*), ethylene response factor 05 (*AtERF05*), *Arabidopsis* dehydration-responsive element-binding protein 1B (*AtDREB1B*), zinc finger protein (*AtZAT7*), and myb domain protein 44 (AtMYB44) were analyzed. * and ** represent significant differences from the control at values of P < 0.05 and P < 0.01, respectively, as determined by Student’s *t*-test.

10.7717/peerj.10524/supp-4 Supplemental Information 4 Primers used in this study.

10.7717/peerj.10524/supp-5 Supplemental Information 5 The basic information of *ClHSP22.8* in watermelon.

10.7717/peerj.10524/supp-6 Supplemental Information 6 Raw data used in qRT-PCR analyses and Figs. 2A, 3A, 3B, 4A, 5 and 6.

## Additional Information and Declarations

### Data Availability

The following information was supplied regarding data availability: The raw data are available as Supplemental Files.
