# Supplementary figures and images for: A heat-shock 20 protein isolated from watermelon (ClHSP22.8) negatively regulates the response of Arabidopsis to salt stress via multiple signaling pathways
Source: PeerJ. 2021 Mar 1;9:e10524. doi: 10.7717/peerj.10524 (PMC7931717; doi:10.7717/peerj.10524)

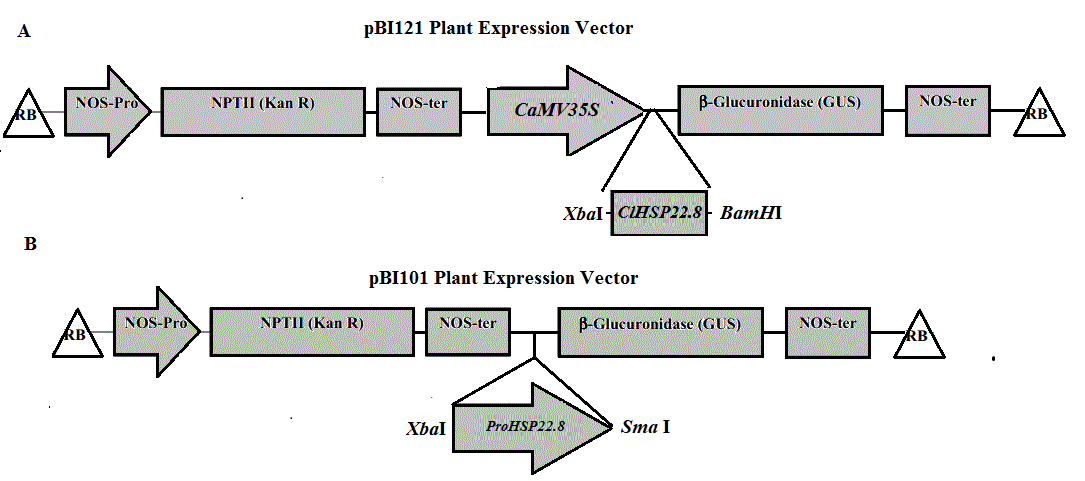

Supplement: Supplemental Information 1 — (A) p35S:: ClHSP22.8/p35S-KanR. The ClHSP22.8 gene (coding sequence only), and a resistance gene, kanamycin (KanR), were both under the control of the CaMV35S promoter. RB, right border; LB, left border (B) Schematic diagram of the ProClHSP22.8::GUS-PBI101 vector. [file peerj-09-10524-s001.png]

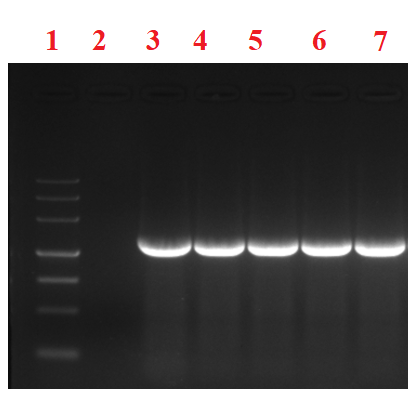

Supplement: Supplemental Information 2 — Lanes 1 to 7 indicate the DNA marker, WT, p35S::ClHSP22.8 vector, and OE22.8-1 to OE22.8-4, respectively. [file peerj-09-10524-s002.png]

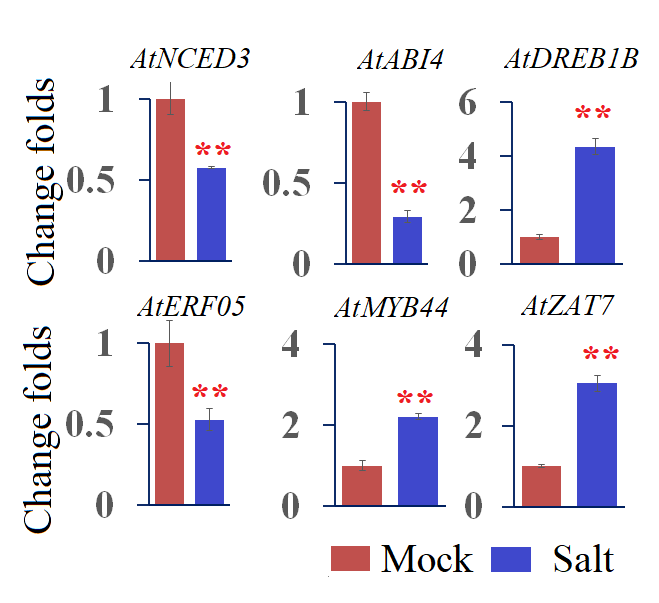

Supplement: Supplemental Information 3 — The expression patterns of Arabidopsis 9-cis epoxycarotenoid dioxygenase 3 (AtNCED3), ABA insensitive 4 (ABI4), ethylene response factor 05 (AtERF05), Arabidopsis dehydration-responsive element-binding protein 1B (AtDREB1B), zinc finger protein (AtZAT7), and myb domain protein 44 (AtMYB44) were analyzed. * and ** represent significant differences from the control at values of P < 0.05 and P < 0.01, respectively, as determined by Student’s t-test. [file peerj-09-10524-s003.png]
